# Legionella on board trains: effectiveness of environmental surveillance and decontamination

**DOI:** 10.1186/1471-2458-12-618

**Published:** 2012-08-07

**Authors:** Gianluigi Quaranta, Sara Vincenti, Anna Maria Ferriero, Federica Boninti, Romina Sezzatini, Cinzia Turnaturi, Maria Daniela Gliubizzi, Elio Munafò, Gianluca Ceccarelli, Carmelo Causarano, Massimo Accorsi, Pasquale Del Nord, Walter Ricciardi, Patrizia Laurenti

**Affiliations:** 1 Institute of Hygiene, Università Cattolica del Sacro Cuore, L.go F. Vito 1, 00168, Rome, Italy; 2 RFI (Italian Railway) Health Service, Via F. A. Pigafetta 3, 00154, Rome, Italy; 3 Trenitalia Environmental Work Quality and Safety, Via F. A. Pigafetta 3, 00154, Rome, Italy

## Abstract

**Background:**

*Legionella pneumophila* is increasingly recognised as a significant cause of sporadic and epidemic community-acquired and nosocomial pneumonia. Many studies describe the frequency and severity of *Legionella* spp. contamination in spa pools, natural pools, hotels and ships, but there is no study analysing the environmental monitoring of *Legionella* on board trains. The aims of the present study were to conduct periodic and precise environmental surveillance of *Legionella* spp. in water systems and water tanks that supply the toilet systems on trains, to assess the degree of contamination of such structures and to determine the effectiveness of decontamination.

**Methods:**

A comparative pre-post ecological study was conducted from September 2006 to January 2011. A total of 1,245 water samples were collected from plumbing and toilet water tanks on passenger trains. The prevalence proportion of all positive samples was calculated. The unpaired *t*-test was performed to evaluate statistically significant differences between the mean load values before and after the decontamination procedures; statistical significance was set at *p* ≤ 0.05.

**Results:**

In the pre-decontamination period, 58% of the water samples were positive for *Legionella*. Only *Legionella pneumophila* was identified: 55.84% were serogroup 1, 19.03% were serogroups 2–14 and 25.13% contained both serogroups. The mean bacterial load value was 2.14 × 10^3^ CFU/L. During the post-decontamination period, 42.75% of water samples were positive for *Legionella* spp.; 98.76% were positive for *Legionella pneumophila*: 74.06% contained serogroup 1, 16.32% contained serogroups 2–14 and 9.62% contained both. The mean bacterial load in the post-decontamination period was 1.72 × 10^3^ CFU/L. According to the *t*-test, there was a statistically significant decrease in total bacterial load until approximately one and a half year after beginning the decontamination programme (*p* = 0.0097).

**Conclusions:**

This study indicates that systematic environmental surveillance could be a useful approach for assessing the risk of exposure to *Legionella* bacteria, which still represents a public health threat. According to the study results, an environmental surveillance programme, followed by decontamination procedures where necessary, would decrease the total bacterial count, protecting the health of travellers and workers.

## Background

*Legionella pneumophila* is increasingly recognized as a significant cause of sporadic and epidemic community-acquired and nosocomial pneumonia.

In recent years, our understanding of Legionnaires’ Disease (LD) has improved substantially and new diagnostic and treatment strategies have been introduced [1].

To share knowledge and monitor trends of LD across Europe, the European Working Group for *Legionella* Infections (EWGLI) was established in 1986 to better protect the health of travellers by improving the detection and control of infection sources in European countries [2].

Members of EWGLI established a European surveillance scheme for travel-associated infections in 1987; this surveillance scheme, named EWGLINET in 2002, is the EU’s surveillance network dedicated to collecting data on cases of LD in the EU, including travel-associated LD (TALD) [3,4].

Since April 2010, the programme has also been coordinated by European Centre for Disease Prevention and Control (ECDC), and the name of the scheme has changed to the European Legionnaires’ Disease Surveillance Network (ELDSNet) [4].

In the beginning of July 2002, the European Guidelines for Control and Prevention of Travel Associated LD [5] were introduced in an attempt to standardise the investigation of clusters in EWGLINET countries and their Ministers of Health were asked to support the use of these guidelines.

In 2009, the EWGLINET surveillance scheme reported a total of 818 cases (794 confirmed and 24 presumptive) of travel-associated LD with onset of illness, which is a decrease of 52 cases compared with 2008 and is 129 fewer than in 2007. The countries that reported the most cases by country of residence were the UK (n = 173), Italy (n = 169) and the Netherlands (n = 109). These three countries have consistently reported the greatest number of cases to EWGLINET for several years [4].

**Figure 1:**
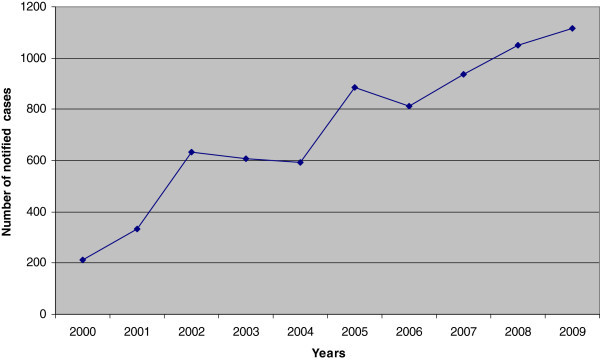
Cases of Legionellosis notified in Italy, 2000–2009 (Data source: Italian Ministry of Health).

The Italian National Surveillance System has reported that, in Italy, the incidence of Legionellosis has been increasing in the last ten years despite a slightly decreasing number of notified cases in the time periods from 2002–2004 and 2005–2006. In 2009, 1,200 cases of Legionellosis were diagnosed and reported (1,146 confirmed and 54 presumptive), with an incidence proportion of twenty cases per million (Figure 1) [6].

In Italy, in 2009, a total of 281 TALD cases, including both Italian and foreign tourists, were reported to the National Health Institute [6]. Among these, 178 cases of legionellosis concern to Italian Tourists. The 86% of them stayed at the hotels, the 5.6% stayed at camping and the 8,4% in other structures. The 10% of 178 cases have travelled in foreign locations.

Many studies describe the frequency and strength of *Legionella* spp. contamination in spa pools, natural pools, hotels and ships [7-17], but no study has been conducted that addresses the environmental monitoring of *Legionella* on board trains.

To protect the health of travellers and workers, the Health Service of Italian Railways and the Hygiene Institute of the Catholic University in Rome established for the first time an environmental surveillance programme to evaluate the degree of contamination on trains and the effectiveness and length of water tank decontamination.

We have examined air conditioning plants and toilet water tanks, which are the primary sources of biological agents. The air conditioning plants on board Trenitalia trains are planned and built to avoid condensed water stagnation. Water is directly drained out of the trains by canalisations that prevent microorganism proliferation. Passenger trains contain water tanks made of steel or resin that supply hygienic services by falling water and they may have a varied capacity (from 200 to 1,800 litres) [18].

## Methods

A comparative pre-post ecological study was conducted from September 2006 to January 2011.

### Water sampling and microbiological analysis

Plumbing and toilet water tanks on passenger trains were examined.

Two samples were collected from every sampling site at a single time point: one sample was harvested immediately after the tap was switched on and without flaming for qualitative evaluation of the *Legionella* species and serogroup and the second sample was collected for quantitative determination (*Legionella* CFU/L of water) after the water ran for at least 5–10 minutes, which is more representative of the water flowing in the system. At least 6 to 12 water samples were collected weekly.

One litre of water was collected in sterile bottles with 0.01% sodium thiosulphate to neutralise any residual chlorine; water samples were transported in a suitable cool box, protected from direct light and processed within four hours after collection.

Microbiological detection of *Legionella* spp. was conducted following the methods described in the “Italian Guidelines for Legionellosis prevention and control” [19,20].

Ten millilitres of the sample were first put into a sterile screw-capped Falcon tube, and then, a one-litre sample of water was concentrated by filtration on a 0.20 μm pore-diameter cellulose membrane filter.

The membranes were aseptically removed and placed into the sterile screw-capped Falcon tube previously filled with 10 ml of water sample collected; the material collected onto the membrane filter was vortex-mixed continuously for 2 min.

Ten millilitres of the sample was divided into two aliquots of 5 ml each; one aliquot was incubated at 50°C in a water bath for 30 minutes (treated sample) and the second aliquot remained at room temperature (non-treated sample).

Next, 0.1 ml of the treated and non-treated samples was spread on a 90 mm Petri dish containing GVPC-selective medium (glycine, polymyxin B, vancomycin and cycloheximide). These plates were incubated at 37°C in a humidified atmosphere with 2.5% CO_2_. Plates were evaluated for a maximum of 14 days before reporting them negative.

Suspected colonies that displayed a distinctive surface with an iridescent and faceted cut glass appearance were counted from each sampling. Gram staining was performed on the suspected colonies; weakly staining, gram-negative bacilli were observable. Suspicious colonies were sub-cultured both onto buffered charcoal yeast extract (BCYE) agar with cysteine and charcoal yeast extract agar (CYE Agar Base – Oxoid) for verification.

These plates were incubated at 37°C in a humidified atmosphere with 2.5% CO_2_ for ≥2 days.

The isolated colonies growing only on BCYE, but that did not grow on CYE, were determined to be *Legionella* colonies. Subsequently, these colonies were serotyped by the agglutination *Legionella* Latex Test (Oxoid) that provides separate identification of *L. pneumophila* serogroup 1, *L. pneumophila* serogroups 2–14 and other species of *Legionella* spp. that have been implicated in human disease: *L. longbeachae* 1 and 2, *L. bozemanii* 1 and 2, *L. dumoffii*, *L. gormanii*, *L. jordanis*, *L. micdadei* and *L. anisa.*

According to the Italian Guidelines [19], quantitative evaluation (CFU/L) was performed considering the following items: the count of colonies growth in each plate, the concentration of the original sample and the dilutions successively made.

### Water supply on board trains and environmental decontamination procedures

The water distributed in the toilets of passenger cars, both for the washbasin and for sanitary services, must have, at the time of supply, the characteristics required for human consumption. Because of the characteristics of hydraulic systems on board, these requirements cannot be fully insured, so water is labelled "no drinking".

Passenger railway cars are fitted with steel or resin tanks in which the total volume of water is variable depending on the type of car (ranging from 200 to 1,800 litres).

The water tanks are located under the car’s roof and the water flows into the toilets by gravity.

Tanks are refilled at stations or through installation maintenance by jet spouts placed on side rails and discharge takes place through a special tap below the wagon.

When *Legionella* bacterial load was greater than 10^3^ CFU/L, decontamination procedures were performed [19,20]. Decontamination was completed in September 2008 (pre-decontamination step = September 2006-September 2008), so October 2008 is considered the beginning of the post-decontamination period.

The measures of supply reservoir and pipeline decontamination involve chemical de-scaling with 20% acetic acid and hyper-chlorination with 20 mg/l for 2 hours or 50 mg/l for 1 hour [19,20], followed by washing to reach 0.2 mg/l of free residual chlorine concentration, or even replacement in limited cases of obvious damage.

### Statistical methods

The prevalence proportion of all positive samples was calculated; the following bacterial load values were adopted to perform a statistical analysis: <100 CFU/L, 100–999 CFU/L, 1,000-9,999 CFU/L, >10,000 CFU/L.

The unpaired *t*-test was performed to evaluate statistically significant differences between the mean load values before and after the decontamination procedures; statistical significance was set at *p* ≤ 0.05.

## Results

The overall number of water samples collected from different trains and analysed from September 2006 to January 2011 was 1,245 (Table 1). Of those, 679 were collected during the pre-decontamination period.

**Table 1:** Results of the microbiological analysis performed on the water samples collected

	**Pre-decontamination period**	**Post-decontamination period**	**Total**
*Number of water samples analysed*	679 (54.538% of total samples analyzed)	566 (45.462% of total samples analyzed)	1245
*Number of samples positive for Legionella* spp.	394 (58.027%)	242 (42.756%)	636
*Number of samples positive for L. pneumophila* 1	220 (55.838%)	177 (73.140%)	397
*Number of samples positive for L. pneumophila* 2 -14	75 (19.036%)	39 (16.116%)	114
*Number of samples positive for L. pneumophila* 1 and 2 -14	99 (25.127%)	23 (9.504%)	122
*Number of samples positive for Legionella other* species	0 (0.000%)	3 (1.240%)	3
*Number of samples with* load values < 100 CFU/L	69 (17.513%)	14 (5.785%)	83
*Number of samples with* load values 100–999 CFU/L	155 (39.340%)	121 (50.000%)	276
*Number of samples with* load values 1,000-9,999 CFU/L	138 (35.025%)	85 (35.124%)	223
*Number of samples with* load values ≥ 10,000 CFU/L	32 (8.122%)	22 (9.091%)	54

In the pre-decontamination period, 58% of water samples were positive for *Legionella* and only *L. pneumophila* was identified; 55.84% contained serogroup 1, 19.03% contained serogroups 2–14 and 25.13% contained both serogroups.

The mean bacterial load value was 2.14 × 10^3^ CFU/L; 17.51% of positive samples had a load value < 100, 39.34% ranged from 100 to 999, 35.03% ranged from 1,000 to 9,999 and 8.12% were > 10,000 CFU/L.

During the post-decontamination period, 566 water samples were collected and analysed: 42.75% were positive for *Legionella* and *Legionella pneumophila* was identified in 98.76% of the positive samples; 74.06% contained serogroup 1, 16.32% contained serogroups 2–14 and 9.62% contained both serogroups.

The mean bacterial load of positive samples in the post-decontamination period was 1.72 × 10^3^ CFU/L: 5.79% had a load value < 100, 50% were between 100 and 999, 35.12% were between 1,000, and 9,999 and 9.09% were > 10,000 CFU/L.

The unpaired *t*-test was performed to compare post-decontamination results to pre-decontamination levels; there was a statistically significant decrease in total bacterial load after January 2009: *p* = 0.0309 (Table 2). The statistically significant decrease in total bacterial load continued until March 2010 (*p* = 0.0097), confirming the intervention’s effectiveness until approximately one and a half years after beginning the decontamination period.

**Table 2:** **Effectiveness of decontamination on*Legionella*bacterial loading**

**Month**	**Mean of load value (CFU/l) (post-decontamination period)**	**Mean of bacterial load in natural logarithmic numbers**	**t**	***p***
October 2008	992.2414	6.9000	0.6591	0.2550
November 2008	583.8235	6.3696	1.1843	0.1183
December 2008	397.0000	5.9839	1.6083	0.0541
January 2009	320.1613	5.7688	1.8698	0.0309
February 2009	295.1282	5.6874	2.1259	0.0169
March 2009	306.3265	5.7247	2.3673	0.0091
April 2009	315.3801	5.7538	2.5402	0.0056
May 2009	295.0256	5.6871	2.7428	0.0031
June 2009	417.9909	6.0355	2.7060	0.0035
July 2009	498.4615	6.2115	2.6624	0.0039
August 2009	554.6850	6.3184	2.6735	0.0038
September 2009	592.1561	6.3838	2.6817	0.0037
October 2009	753.6237	6.6249	2.4677	0.0069
November 2009	752.5163	6.6234	2.5500	0.0055
December 2009	748.6751	6.6183	2.6023	0.0047
January 2010	726.0479	6.5876	2.7144	0.0034
February 2010	934.4538	6.8400	2.3195	0.0103
March 2010	962.5000	6.8695	2.3432	0.0097
April 2010	1369.3270	7.2221	1.3341	0.0912
May 2010	1314.9530	7.1816	1.4680	0.0712
June 2010	1521.4860	7.3274	1.0917	0.1376
July 2010	1482.9550	7.3018	1.1885	0.1174
August 2010	1707.7110	7.4429	0.7837	0.2167
September 2010	1697.6230	7.4370	0.8044	0.2107
October 2010	1684.2690	7.4291	0.8432	0.1996
November 2010	1635.7890	7.3999	0.9556	0.1697
December 2010	1719.8530	7.4500	0.8037	0.2109
January 2011	1721.6250	7.4510	0.8141	0.2079

[In Table 2 are reported the results of the unpaired t-tests performed to compare the mean value of the bacterial load in the pre-decontamination period (grand mean: 2142.7840 CFU/L – 7.6698 in logarithmic value) with the ones of the post-decontamination period].

## Discussion

Several studies describe the frequency of *Legionella* spp. contamination in spa pools, natural pools and hotels [7-9]. Cases of LD are also well documented among cruise ship passengers [9-17]. To date, this is the first study concerning the environmental monitoring of *Legionella* on board trains.

This study demonstrates the necessity for periodic evaluation on trains to assess the potential contamination of *Legionella* spp., with the aim to drive decontamination efforts in the absence of specific guidelines. We hypothesized that an environmental surveillance programme, followed by decontamination procedures when necessary, would decrease the total bacterial load, thereby protecting the health of travellers and workers.

This study demonstrated a statistically significant decrease in total bacterial load after decontamination procedures. The *t*-test indicated a decrease in bacterial load since October 2008, with statistical significance from January 2009 to March 2010, suggesting that a disinfection treatment performed every one and a half years could reduce environmental contamination.

Moreover, this study demonstrated a decrease in *Legionella*-positive samples after decontamination procedures: 58% in the pre-decontamination period and 42.75% in post one were positive for *Legionella.* Although there was a decrease in the total *Legionella*-positive samples, we observed an increase of *Legionella* serogroup 1 (55.84% pre- vs. 74.06% post-decontamination), which is responsible for approximately 70% of *Legionella* infections in Europe [21]. This increase could have been due to the development of a protective biofilm, especially when load values were high. As demonstrated in this study, the disinfection treatments were more effective when the bacterial load was lower (17.51% of positive samples with a load value < 100 CFU/L in pre- vs. 5.79% in post-decontamination).

Even if hyper-chlorination of the water is consistently performed, this approach is particularly appropriate for the treatment and removal of planktonic cultures of *L. pneumophila*, but remains ineffective against sessile communities of the bacterium [22].

This study suggests that the resistance to chlorination of *L. pneumophila* serogroup 1 could be due to the biofilm that facilitates a higher tolerance to the disinfectant. In fact, we observed limestone deposits in different water tanks in which the biofilm could have been developed. Environmental strains could develop a resistance mechanism to chlorine-based disinfection treatments, so these decontamination procedures are not completely appropriate for removing the bacterium from plumbing and tanks. As these procedures do not lead to a definitive solution to the problem, we suggest that mechanical cleaning could increase the effectiveness and length of chemical decontamination procedures, thereby reducing bacterial load values and increasing the amount of time that the water can be considered safe.

We aimed to highlight that environmental surveillance represents an evidence-based approach for evaluating the contamination of the water supply on board trains. This work stresses the effectiveness of decontamination and, above all, provides information about the required frequency of disinfection procedures to achieve a statistically significant decrease in total bacterial load.

Periodic environmental surveillance could be a useful approach for assessing the risk of exposure to *Legionella* bacteria, which still poses a threat to public health. *Legionella* spp. are opportunists par excellence and are therefore especially dangerous for vulnerable individuals (e.g., older people, current tobacco smokers, immune-compromised or immune-suppressed persons, patients with chronic degenerative diseases and transplanted people).

Our purpose is to ensure the safety of the passengers and the workers on board trains by defining proper preventive measures to be implemented with an established frequency.

The primary limitation of this study concerns the use of the *t*-test; a paired *t*-test was not performed because this study focused on the general trend of contamination/decontamination on board trains as opposed to a specific sampling site.

Moreover in this study we analyzed only environmental data because we were given the responsibility of doing only the environmental surveillance; in fact, clinical surveillance was carried out by other institutions and, up to date, we have no information about cases of Legionellosis linked to our environmental data.

The lengthy time period of the environmental surveillance and the large number of water samples collected and analysed represent a point of strength in this study.

## Conclusions

Public health programmes should focus on reducing the risk for LD among travellers.

In addition to cruise ships and ferries [16], the routine monitoring of water on board trains must be considered an essential measure of primary prevention for guaranteeing water safety and, consequently, to avoid cases of LD. For these reasons, the drafting of guidelines for LD prevention on public transportation may be necessary to protect the health of travellers and workers. According to our experience, it is necessary to pay close attention to water quality on board trains, especially after decontamination procedures to prevent bacterial colonization.

Although the eradication of *Legionella* spp. in plumbing and water tanks is difficult to achieve, our approach can be considered an evidence-based method for assessing and managing *Legionella* infection risk on trains.

The air conditioning plants on board Trenitalia trains are designed to avoid air conditioner condensation drip. The particular characteristics of water supply facilities on trains, which work by gravity at a low pressure, and the lack of showers reduce the possibility of the formation of aerosol, which is potentially dangerous for Legionellosis. Nevertheless, Italian Railways have carried out the present study in collaboration with Catholic University in Rome and the positive results may improve future train sanitation following the practical measures described in detail in this article.

## Abbreviations

CFU: Colony forming unit; CFU/L: Colony forming unit/litre.

## Competing interests

The authors declare that they have no competing interests.

## Authors' contributions

GQ participated in the design of the study, contributed to draft the manuscript and performed the statistical analysis. SV performed the microbiological analysis, conceived of the study and helped to draft the manuscript. AMF conceived of the study and helped to manage the database and to draft the manuscript. FB performed the microbiological analysis. RS performed the microbiological analysis. CT performed the microbiological analysis. MDG helped to manage the database and draft the manuscript. GC,CC, MA and PDN conceived of the study and participated in its design and coordination. EM conceived of the study and participated in its design and coordination, allowing the surveillance on board train. WR conceived of the study and participated in its design and coordination. PL conceived of the study, participated in its design and coordination, carried out the surveillance and helped to draft the manuscript. All authors read and approved the final manuscript.

## Pre-publication history

The pre-publication history for this paper can be accessed here:

http://www.biomedcentral.com/1471-2458/12/618/prepub
